# Association Between Birth Outcomes and Gestational Weight Gain Among Forcibly Displaced Rohingya and Nearby Host Community, in Cox’s Bazar, Bangladesh

**DOI:** 10.3390/life15111773

**Published:** 2025-11-19

**Authors:** Shakil Ahamed, Elisa Ugarte, Mahbub Elahi, Eamam Hossain, Sajjadur Rahman, Kazi Istiaque Sanin, Abir Dutta, Goutam Kumar Dutta, Alice J. Wuermli, Fahmida Tofail

**Affiliations:** 1 Maternal and Child Nutrition Department, Nutrition Research Division, International Centre for Diarrheal Disease Research, Bangladesh (icddr,b), Mohakhali, Dhaka 1212, Bangladesh; mahbub.elahi@icddrb.org (M.E.); abir.dutta@icddrb.org (A.D.);; 2 Global Ties for Children, New York University, New York, NY 10012, USA

**Keywords:** gestational weight gain, forcibly displaced Rohingya, pregnancy, birth outcomes, maternal health, neonatal health

## Abstract

Gestational weight gain (GWG) is a critical determinant of maternal and neonatal health, yet its patterns and consequences in displaced populations remain understudied. This study examined the association between GWG and birth outcomes among Forcibly Displaced Rohingya (FDR) women in Cox’s Bazar, Bangladesh. We conducted a longitudinal cohort study from October 2022 to October 2024, enrolling 2888 pregnant women at different stages of pregnancy. Among them, 301 were recruited in the first trimester and followed through the third trimester, with 231 neonatal outcomes recorded within 72 hours of delivery. Overall, 66.8% of women experienced inadequate GWG. Despite the high prevalence of inadequate GWG, mean birth weight (2.79 kg) and mean gestational age at delivery (38.6 weeks) were within favorable ranges. Inadequate GWG was more common in mothers aged 30–39 years (*p* = 0.061) but significantly less common in underweight mothers (*p* = 0.012). GWG was positively associated with neonatal birth weight, length, and weight–length ratio (WLR) Z score, but not with gestational age. After adjusting for confounding factors, inadequate GWG showed a significant independent association with lower birth length (*p* = 0.016). These findings highlight the need for targeted interventions in displaced populations.

## 1. Introduction

Gestational weight gain (GWG) is a critical determinant of maternal and neonatal health outcomes. Adequate GWG supports optimal fetal growth and reduces the risk of complications such as low birth weight and preterm delivery [1]. Conversely, inadequate GWG is associated with heightened risks for adverse outcomes, frequently linked to poor nutrition, psychosocial stress, and restricted access to healthcare services [2,3,4]. These risks are particularly pronounced in forcibly displaced women, who often encounter food insecurity, overcrowded living conditions, economic instability, and disrupted healthcare systems—factors that exacerbate the adverse effects of inadequate GWG [5,6,7].

Evidence suggests that, in resource-limited settings, the prevalence of inadequate GWG is alarmingly high. For example, over 72% of women in Ethiopian refugee camps and nearly 80% of rural women in Northern Ghana failed to achieve the recommended GWG [8,9]. Such deficits are strongly associated with increased risks of preterm birth and low birth weight [10,11]. The interplay of socio-economic determinants, including poverty, limited educational opportunities, and heightened maternal stress, further illustrates the compounded vulnerabilities faced by displaced populations [12]. Community-based strategies such as resource sharing and social support, on the other hand, have been shown to mitigate adverse outcomes [13].

In 2017, the Myanmar crisis forced over 700,000 Rohingya, a Muslim minority group from Rakhine State, to flee to Cox’s Bazar, Bangladesh, where they now live in the world’s largest refugee camps. These individuals, known as forcibly displaced Rohingya or FDR, continue to face statelessness, restricted rights, and limited humanitarian support. However, the relevance and effectiveness of coping mechanisms among FDR women—those uprooted during the 2017 crisis and residing in the overcrowded camps of Cox’s Bazar—remain largely underexplored [13,14]. This gap highlights the urgent need for context-specific research to clarify the relationship between inadequate GWG and birth outcomes in this population.

Birth outcomes such as birth weight, birth length, head circumference, and gestational age at birth are critical indicators of neonatal health and long-term developmental potential. In refugee context these outcomes are strongly influenced by a range of maternal factors, including nutritional status, GWG, age, body mass index (BMI), psychosocial stress, education level, parity, and access to healthcare services [2,12]. Mental health problems in contexts of displacement also contribute significantly to maternal and neonatal outcomes. Studies found that psychosocial stress, particularly among migrant women with insecure residency, was associated not only with higher risks of preterm birth but also with adverse maternal health outcomes, such as hypertensive disorders, gestational diabetes, and increased risk of postpartum depression [15]

Healthcare provider support plays a crucial role in mitigating inadequate GWG; however, evidence suggests that healthcare professionals often fail to deliver effective counseling due to insufficient training and limited resources, while barriers such as time constraints and cultural differences further hinder communication [4,5,15,16,17,18,19]. Consequently, there is a pressing need for culturally sensitive training and context-adapted approaches for healthcare providers working in refugee settings [16].

There are also reports that social support correlates positively with improved maternal and neonatal outcomes [18]. Vulnerable groups such as asylum-seeking women experiencing pregnancy without partner support face higher risks of serious maternal health problems, emphasizing the need for targeted care [12].

This study, therefore, aims to investigate the association between inadequate GWG and birth outcomes among FDR women residing in Cox’s Bazar, Bangladesh. By generating context-specific evidence, it seeks to inform maternal healthcare strategies tailored for displaced populations worldwide.

## 2. Materials and Methods

### 2.1. Study Design

**Figure 1:**
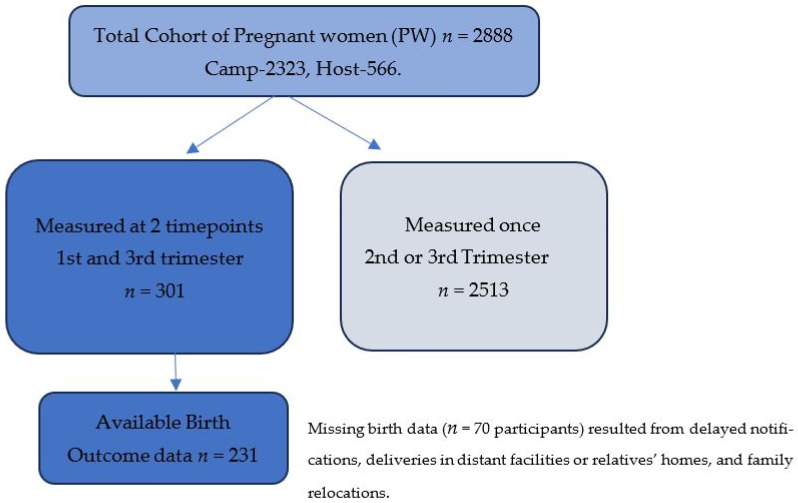
Sample selection Flowchart.

We conducted a longitudinal cohort study and collected data in the Forcibly Displaced Myanmar Nationals (FDMN) camps in Cox’s Bazar, Bangladesh, and surrounding host communities, from February 2023 to March 2024. As part of this study, we enrolled 2888 pregnant women, including 2322 from the camps and 566 from the surrounding host community. Details on study design can be found in our cohort profile paper [20]. For the current analysis, we focused on pregnant women with first and third-trimester gestational weight measurements (*n* = 301, Camp = 227, Host = 74). Among these, 231 women had documented birth outcomes within 72 h of birth. Missing birth-related data was due to delayed birth notifications, deliveries occurring in distant hospitals or relatives’ homes, or changes in the family’s location. Sample selection steps are shown in Figure 1.

### 2.2. Sample Selection and Procedure

A purposive sampling method was used to select participants, ensuring a representative sample of pregnant women across different trimesters. Data collection involved structured interviews, medical records, and physical and biological examinations. Pregnant women who were aged between 13 and 45 years and willing to participate were included in the study. Those with severe chronic diseases or who refused to participate were excluded.

### 2.3. Data Collection Procedure and Tools

Detailed demographic information, clinical history, socio-economic status, and health behaviors were obtained through interviews. Maternal weight, height, and BMI were assessed as indicators of nutritional status. Maternal education was defined as the highest level of formal schooling attained, while parity denoted the number of previous live births. Pregnancy complications were classified as “yes” when a participant was diagnosed with hypertension, gestational diabetes, chronic kidney disease, heart disease, asthma, stroke, any infection, hepatitis, or anemia, as reported by a physician and confirmed through laboratory findings. Access to healthcare encompassed the availability of essential maternal health services within the study setting.

All anthropometric measurements were obtained by trained medical technologists and data collectors using standardized equipment. Body weight was measured with a Tanita HA-650 digital weighing scale, which demonstrated good test–retest reliability (99.99%), intra-observer reliability (99.98% and inter-instrument reliability 99.95%). Height and length were assessed using SECA 213 stadiometers, showing high reliability across measures (inter-observer reliability 99.93%, intra-observer reliability 99.96%, and inter-instrument reliability 99.92%). To ensure accuracy, the instruments were calibrated daily using fixed weights and lengths before data collection. Measurement reliability and inter-observer consistency were routinely monitored throughout the study to maintain the validity of the anthropometric data. Maternal age was grouped into three categories: <20 years, 20–29 years, and 30–39 years (there were no participants ages 40 and above). GWG was categorized using the Institute of Medicine (IOM) guidelines [21] which classify weight gain into three categories: inadequate, adequate, and excessive. This paper will focus on the first two (inadequate and adequate) because excessive gestational weight gain is very uncommon in this population. For analysis, the Z-score of GWG was calculated using the Intergrowth-21 protocol to standardize GWG based on gestational age. Z-score lower than or equal to −1 was considered inadequate GWG, and higher than −1 was considered adequate [22]. Maternal early pregnancy BMI was categorized according to Asian-specific cut-offs adapted from the IOM guidelines: Underweight (<18.5 kg/m^2^), normal weight (18.5–22.9 kg/m^2^), overweight (23.0–27.4 kg/m^2^), and obese (≥27.5 kg/m^2^) [23]. Birth outcomes, including birth weight, birth length, head circumference, and weight-for-length Z-score (WLZ) at birth based on gestational age, were collected through direct measurement. All anthropometric measurements were conducted by trained Rohingya volunteers who received five days of intensive training in infant anthropometry, followed by periodic refresher sessions and close supervision to ensure accuracy. Infant weight was measured using a SECA infant scale (Model 354, Hamburg, Germany) with a precision of up to 1 g, while birth length was measured to the nearest millimeter using standardized wooden infantometers according to recommended procedures. Equipment was calibrated daily with standard weights, and inter-observer reliability checks were routinely performed to maintain measurement consistency and validity. The reliability of birth anthropometric measurements in this study was high (coefficient = 99.98%). Additional birth-related data, such as the place of delivery, complications, and gestational age at birth, were also recorded.

### 2.4. Statistical Analysis

Descriptive statistics were applied to summarize sample characteristics. *t*-tests and logistic regression analyses were used to identify factors associated with inadequate gestational weight gain (GWG), including maternal body mass index (BMI), age, and psychosocial stress. The model controlled for key confounders such as maternal education, parity, and access to healthcare.

To assess the association between GWG and primary birth outcomes—namely birth weight, birth length, and gestational age—both correlation and regression analyses were conducted. Variable selection was guided by evidence from prior epidemiological and perinatal research. Unadjusted and adjusted linear and logistic regression models were employed, with adjusted models accounting for potential confounders such as socioeconomic status and prenatal care. Socioeconomic status was evaluated based on household income, asset ownership, and living conditions, while prenatal care captured the number and timing of antenatal visits received during pregnancy. Following the INTERGROWTH protocol, fetal growth Z-scores were derived according to gestational age at birth and examined in relation to maternal GWG Z-scores to elucidate the role of GWG as a determinant of fetal growth and neonatal health.

### 2.5. Ethical Considerations

This study (Protocol number) received ethical approval from the Institutional Review Boards (IRB Number: PR-22064) of icddr,b and New York University Institutional Review Board (IRB-FY2021-4875). Informed written consent was obtained from all participants, with clear explanations of the study’s purpose, procedures, and confidentiality measures. Participants were assured that their data would remain confidential and that they could withdraw at any stage without consequence. Those with identified health issues were referred for appropriate medical care within the camp.

## 3. Results

As shown in Table 1, 301 pregnant women were categorized by gestational weight gain (GWG) status. Among those with inadequate weight gain (*n* = 201, 66.78%), and maternal age was significantly associated with weight gain status (*p* = 0.018). Education level did not differ significantly between groups (*p* = 0.522). Most women had either madrassa education (30.85% vs. 35%) or more than primary schooling (38.81% vs. 34%) in both groups. Parity also showed no significant difference (*p* = 0.507), with nearly equal proportions of nulliparous, primiparous, and multiparous women in both categories. Early-pregnancy BMI, however, was significantly associated with weight gain (*p* = 0.007). There were very few pregnancy complications, only 17 (5.65%) pregnant mother reported any type of pregnancy complication or disease, and more occurred in the inadequate GWG group. The majority of women in both groups had normal BMI. Access to toilet and bathing facilities was similar across both groups (*p* = 0.778 and *p* ≥ 0.999, respectively).

In multiple logistic regression analysis shown in Table 2, factors associated with inadequate weight gain among 301 pregnant women were assessed. Mothers aged 30–39 years had higher odds of inadequate weight gain compared to those aged below 20 years (AOR: 3.96; 95% CI: 1.03–19.9; *p* = 0.061). Women with underweight BMI (<18.5 kg/m^2^) had significantly lower odds of inadequate weight gain compared to those with normal BMI (AOR: 0.40; 95% CI: 0.19–0.81; *p* = 0.012). Pregnant women who reported complications or diseases had 1.89 times higher odds of inadequate weight gain (AOR: 1.89; 95% CI: 0.55–8.79; *p* = 0.356), although this was not statistically significant. Other factors—including education level, parity, overweight/obese BMI, and access to toilet or bathing facilities—were not significantly associated with inadequate weight gain after adjusting for other covariates in the model.

Among the 231 newborns with available data shown in Table 3 the majority were healthy at birth (214; 92.64%), while (9; 3.90%) were reported ill and (8; 3.46%) died. Differences in demographic characteristics between documented and undocumented birth outcome groups were minimal (see Appendix A Table A1). Most deliveries occurred in hospitals (131; 56.71%), with normal vaginal delivery being the predominant mode (199; 86.52%). Birth complications were reported in more than half of the cases (120; 51.95%). Nearly all births were term (221; 95.67%), with (6; 2.60%) preterm and (4; 1.73%) post-term. Low birth weight was observed in (42; 18.83%) newborns. The mean birth weight was 2.8 kg (±0.38), mean birth length (48.6 cm ± 2.04), mean gestational age (38.8 weeks ± 3.12), and mean WLZ (−1.4 ± 0.96).

**Figure 2:**
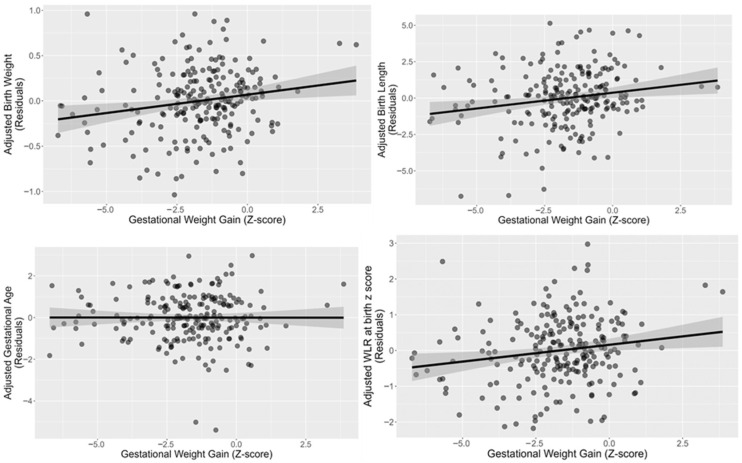
Scatterplots showing the relations between adjusted Birth weight, length, gestational age, and fetal growth WRL Z score with gestational Weight Gain Z score (*n* = 223).

In Figure 2 Multiple linear regression analyses showed that GWG had a significant impact on key birth outcomes. After adjustment for maternal age, education, parity, pregnancy complications and pre-pregnancy BMI, GWG (z-score) was positively associated with birth weight (Adjusted β = 0.04; 95% CI: 0.01–0.07; *p* = 0.005), birth length (Adjusted β = 0.23; 95% CI: 0.07–0.39; *p* = 0.006), and fetal growth WRL z-scores (Adjusted β = 0.10; 95% CI: 0.02–0.18; *p* = 0.010). In contrast, no significant association was found between GWG and gestational age at birth (*p* > 0.05).

When we analyzed the association of inadequate weight gain (as a dichotomous factor) with birth outcomes-shown in Table 4, we found a statistically significant association with birth length only. Mothers with inadequate gestational weight gain gave birth to infants with a lower mean birth length (48.4 ± 2.16 cm) compared to those with adequate weight gain (49.1 ± 1.72 cm, *p* = 0.0162). However, no significant associations were observed with birth weight (*p* = 0.1282), gestational age at birth (*p* = 0.1722) and WRL Z score at birth (*p* = 0.217) although all outcomes were slightly higher in the adequate weight gain group.

## 4. Discussion

In our study, we found that nearly 67% of mothers experienced inadequate GWG. Mothers aged 30–39 were most likely to have inadequate weight gain, while those with low early pregnancy BMI were significantly more likely to gain adequately. Interestingly, demographic and pathological factors such as maternal education, parity, and access to toilets and bathing facilities and pregnancy complications did not demonstrate any significant impact on GWG.

Gestational weight gain (GWG) was positively associated with birth weight, birth length, and weight-for-length z-scores, but showed no association with gestational age at birth. When GWG was categorized according to INTERGROWTH 21 and associations were tested, inadequate GWG was significantly associated with reduced birth length only.

These findings reinforce the critical influence of maternal health parameters like age, BMI on both maternal weight gain and neonatal outcomes, as previously reported [24,25,26]. Maternal age, in particular, plays a complex role. Older mothers, especially those aged 30–39, are more prone to inadequate GWG due to metabolic and lifestyle factors such as reduced basal metabolic flexibility, lower appetite regulation, and greater likelihood of sedentary behavior compared with younger women [24,27]. Psychosocial factors may also play a role—older women often worry about gaining excessive weight and some may enter pregnancy with conditions such as hypertension or diabetes that require dietary restrictions. cultural beliefs and taboos also influence maternal diet, as many women think that eating more will lead to a larger baby and complications during delivery [25,26].

Regarding BMI, our data revealed that women with lower pre-pregnancy BMI were more capable of achieving adequate GWG. This observation underscores the compensatory physiological mechanisms present in underweight women. In a study it was shown that, intervention for increasing appetite, more efficient nutrient absorption, and adaptive hormonal responses such as elevated ghrelin and leptin sensitivity promote weight gain when starting pregnancy from a nutritionally disadvantaged state [24]. In contrast, sometimes women with higher pre-pregnancy BMI may consciously restrict food intake due to advice or personal concerns about obesity-related risks, and they may also experience metabolic resistance to weight gain due to insulin resistance and altered lipid metabolism [26,27]. However, in this particular population, obesity was rare. Moreover, almost all pregnant women received the same type of nutritional support regardless of BMI both camp and host community, minimizing systemic differences in care. Instead, variations in personal dietary choices and actual food intake are more plausible contributors to the differences observed.

The association between GWG and neonatal size outcomes in our analysis highlights the direct role of maternal nutritional status in shaping fetal growth trajectories. Adequate weight gain enhances placental blood supply and nutrient transfer, improving fetal fat deposition and skeletal growth [27,28]. Our finding that gestational age was unaffected by GWG reinforces the idea that weight gain primarily affects intrauterine growth, while parturition timing is governed more by endocrine triggers, infection, or inflammatory pathways [28,29]. Thus, maternal nutrition appears to determine how well a baby grows in utero, rather than when the baby is delivered.

A particular finding of our study was the reduction in birth length in infants born to mothers with inadequate GWG. Birth length is considered a marker of skeletal growth and long-term growth potential, making it a critical indicator beyond birth weight alone. Mechanistically, insufficient GWG may limit micronutrient intake (e.g., calcium, vitamin D, zinc), which are essential for endochondral ossification and bone elongation [30,31]. Chronic maternal undernutrition also downregulates Insulin like Growth Factor-1 signaling at the placental–fetal interface, impairing chondrocyte proliferation and leading to restricted linear growth [32]. This is particularly relevant in our study setting, where intergenerational stunting is already prevalent; our findings suggest that inadequate maternal weight gain could be a critical upstream driver of this cycle.

Interestingly, demographic and pathological characteristics such as maternal education, parity, sanitation access, and pregnancy complications did not significantly influence GWG or neonatal outcomes. While previous studies have shown protective effects of maternal education on pregnancy nutrition and outcomes [33], in our context, widespread nutritional vulnerability may overwhelm the advantages conferred by education or hygiene. Even educated mothers may face household-level food insecurity or limited dietary diversity, restricting their ability to act on knowledge. Similarly, parity did not differentiate outcomes in our study; this may be because both primiparous and multiparous women are exposed to the same physiological stress of food scarcity, which could mask differences usually observed in better-resourced populations [34]. These findings suggest that in resource-limited settings environmental and nutritional determinants may dominate over social gradients.

Despite these important insights, several limitations must be acknowledged. The absence of dietary intake data restricted our ability to identify specific nutrient deficiencies contributing to inadequate GWG. Similarly, the lack of serial ultrasonography limited assessment of intrauterine growth restriction patterns in relation to maternal weight gain trajectories. Recall bias may also have influenced self-reported information, and the cross-sectional nature of some measures constrains causal interpretation. In addition, differences between camp and host mothers were not observed due to limited variability and the smaller sample size in the host group. Finally, the findings may not be generalizable beyond the studied camp and host populations. However, the strengths of our longitudinal approach and the use of standardized anthropometric measures provide robust evidence regarding maternal and fetal health dynamics in this vulnerable population.

## 5. Conclusions

Our findings underscore the significant association between early-pregnancy BMI and inadequate gestational weight gain (GWG), as well as the link between inadequate GWG and adverse birth outcomes. While demographic factors appeared to have minimal influence, maternal physiological indicators emerged as critical determinants of both maternal health and neonatal outcomes. These insights highlight the need for targeted interventions that provide nutritional counseling and support for mothers particularly those from vulnerable demographic groups to optimize pregnancy health and promote improved fetal growth and neonatal outcomes.

## Figures and Tables

**Table 1:** The demographic characteristics of 301 pregnant women, categorized by gestational weight gain status.

Characteristic	Inadequate Weight Gain (z Score ≤ −1) *n* = 201 ^1^ (66.77%)	Adequate Weight Gain (z Score > −1) *n* = 100 ^1^ (33.22%)	*p*-Value
Mother Age (Year)			0.018 ^2^
below 20	106 (52.74)	66 (66.00)	
20–29	72 (35.82)	31 (31.00)	
30–39	23 (11.44)	3 (3.00)	
Education			0.522 ^2^
No School (No class)	11 (5.47)	9 (9.00)	
Madrassa (Religious education)	62 (30.85)	35 (35.00)	
Reached primary (Preschool to class 3)	50 (24.88)	22 (22.00)	
More than primary (More than class 3)	78 (38.81)	34 (34.00)	
Parity			0.507 ^2^
Nulliparous	75 (37.31)	41 (41.00)	
Primiparous	50 (24.88)	28 (28.00)	
Multiparous	76 (37.81)	31 (31.00)	
First trimester (Early Pregnancy) BMI			
Underweight (<18.5 kg/m^2^)	18 (8.96)	22 (22.00)	0.007 ^2^
Normal Weight (18.5–22.9 kg/m^2^)	114 (56.72)	55 (55.00)	
Overweight (23–27.4 kg/m^2^)	31 (15.42)	13 (13.00)	
Obese ≥ 27.5 kg/m^2^	38 (18.91)	10 (10.00)	
Pregnancy Complications and diseases	14(06.97)	3(3.00)	0.160 ^2^
Toilet facility	181 (90.05)	89 (89.00)	0.778 ^2^
Bathing facility	192 (95.52)	95 (95.00)	>0.999 ^3^

^1^ *n* (%), ^2^ Pearson’s Chi-squared test, ^3^ Fisher’s exact test.

**Table 2:** Multiple Logistic Regression for Mothers Inadequate Weight Gain, (*n* = 301).

Characteristic	AOR (95% CI)	*p*-Value
Mother Age (Year)		
below 20	—	
20–29	1.33 (0.65, 2.78)	0.436
30–39	3.96 (1.03, 19.9)	0.061
Education		
No School (No class)	—	
Madrassa (Religious education)	1.44 (0.50, 4.07)	0.494
Reached primary (Preschool to class 3)	1.93 (0.64, 5.70)	0.235
More than primary (More than class 3)	1.96 (0.67, 5.65)	0.212
Parity		
Nulliparous	—	
Primiparous	0.92 (0.47, 1.78)	0.799
Multiparous	0.89 (0.38, 2.07)	0.780
Early pregnancy BMI		
Normal Weight (18.5–22.9 kg/m^2^)	—	
Underweight (<18.5 kg/m^2^)	0.40 (0.19, 0.81)	0.012 *
Overweight (23–27.4 kg/m^2^)	1.01 (0.48, 2.19)	0.983
Obese ≥ 27.5 kg/m^2^	1.39 (0.63, 3.26)	0.434
Pregnancy complications and diseases		
No	—	
Yes	1.89 (0.55, 8.79)	0.356
Toilet facility		
No	—	
Yes	1.14 (0.48, 2.61)	0.754
Bathing facility		
No	—	
Yes	1.05 (0.30, 3.33)	0.937

Significant *p*-values (<0.05) are marked with an asterisk (*), Adjusted for all other variables in the model.

**Table 3:** Characteristics at Birth (*n* = 231).

Characteristic	*n* = 231 ^1^ (100%)
Birth condition	
Healthy	214 (92.64)
Ill	9 (3.90)
Died	8 (3.46)
Birth place	
Home	100 (43.29)
Hospital	131 (56.71)
Birth born type (*n* = 230)	
Normal	199 (86.52)
Cesarean	8 (3.48)
Mechanical support delivery	23 (10.00)
Unknown	1
Birth complication	
No complication	111 (48.05)
Complication	120 (51.95)
Gestational age	
Preterm (<37 week)	6 (2.60)
Normal term (37 to 41 week)	221 (95.67)
Post term (>41 week)	4 (1.73)
Birth weight (*n* = 223)	
Low birth weight	42 (18.83)
Normal	181 (81.17)
Unknown	8
Gestational weight gain	
Inadequate weight gain (z score ≤ −1)	151 (65.37)
Adequate weight gain (z score > −1)	80 (34.63)
	Mean (±SD)
Birth weight (*n* = 223)	2.8 (±0.38)
Unknown	8
Birth length (*n* = 223)	48.6 (±2.04)
Unknown	8
Gestational age at birth	38.8 (±3.12)
Birth WLR z score (*n* = 223)	−1.4 (±0.96)

^1^ *n* (%); Mean (±SD).

**Table 4:** Associations Between Birth Outcomes and Inadequate and Adequate Gestational Weight Gain (*n* = 231).

Characteristic	Inadequate Weight Gain (z Score ≤ −1), *n* = 151 ^1^	Adequate Weight Gain (z Score > −1), *n* = 80 ^1^	*p*-Value
Birth weight	2.8 (±0.40)	2.8 (±0.33)	0.128 ^2^
Unknown	6	2	
Birth length	48.4 (±2.16)	49.1 (±1.72)	0.016 ^2^
Unknown	6	2	
Gestational age at birth	38.6 (±3.73)	39.2 (±1.26)	0.172 ^2^
Birth Z score	−1.5 (±0.94)	−1.3 (±0.98)	0.217 ^2^
Unknown	6	2	

^1^ Mean (±SD), ^2^ Two Sample *t*-test.

## Data Availability

Due to icddr,b data access policy regarding participant-identifying information, the data are available upon request from the Research & Clinical Administration and Strategy (RCAS) at icddr,b.

## Abbreviations

The following abbreviations are used in this manuscript:

OD	Odds Ratio
CI	Confidence Interval
SD	Standard Deviation

## Appendix A

**Table A1:** Missing data with not messing characteristics.

Characteristic	Not Missing at Birth *n* = 231 ^1^ (%)	Missing at Birth *n* = 70 ^1^ (%)	*p*-Value
Mother Age (Year)			0.334 ^2^
Below 20	127 (54.98)	45 (64.29)	
20–29	82 (35.50)	21 (30.00)	
30–39	22 (9.52)	4 (5.71)	
Education			0.516 ^3^
No School (No class)	18 (7.79)	2 (2.86)	
Madrassa (Religious education)	74 (32.03)	23 (32.86)	
Reached primary (Preschool to class 3)	53 (22.94)	19 (27.14)	
More than primary (More than class 3)	86 (37.23)	26 (37.14)	
Parity			0.004 ^2^
Nulliparous	78 (33.77)	38 (54.29)	
Primiparous	61 (26.41)	17 (24.29)	
Multiparous	92 (39.83)	15 (21.43)	
Pre pregnancy BMI			0.994 ^2^
Underweight (<18.5 kg/m^2^)	30 (12.99)	10 (14.29)	
Normal Weight (18.5–22.9 kg/m^2^)	130 (56.28)	39 (55.71)	
Overweight (23–24.9 kg/m^2^)	34 (14.72)	10 (14.29)	
Obese ≥ 25 kg/m^2^	37 (16.02)	11 (15.71)	
Toilet facility	203 (87.88)	67 (95.71)	0.059 ^2^
Bathing facility	219 (94.81)	68 (97.14)	0.533 ^3^

^1^ *n* (%); ^2^ Pearson’s Chi-squared test; ^3^ Fisher’s exact test.
